# Backcasting approach with multi-scenario simulation for assessing effects of land use policy using GeoSOS-FLUS software

**DOI:** 10.1016/j.mex.2019.05.007

**Published:** 2019-05-17

**Authors:** Yuncai Wang, Jiake Shen, Wentao Yan, Chundi Chen

**Affiliations:** a Department of Landscape Architecture, Ecological Wisdom and Practice Research Center, College of Architecture and Urban Planning, Tongji University, China; b Department of Landscape Studies, College of Architecture and Urban Planning, Tongji University, China; c Department of Urban Planning, Ecological Wisdom and Practice Research Center, College of Architecture and Urban Planning, Tongji University, China; d Chongqing Institute of Green and Intelligent Technology, Chinese Academy of Sciences, China

**Keywords:** Backcasting approach with multi-scenario simulation (BAMSS), Cellular Automata model, Conversion cost matrix, Parameter translation, Land use simulation, Policy effectiveness

## Abstract

The method presented is helpful for assessing effects of land use policy and unfolding the action mechanism and implementation effect of policies on land use changes. The backcasting method is based on the re-simulation of the historical real changes of land use. By observing the error between the real and simulated states, adjust the parameters for many times, and a variety of land use scenarios are obtained, and the scenario with the minimum and acceptable error is selected to assess the effects of land use policy represented by its parameters. The method is structured in four sequential stages, including expressing the policy action mechanism as the parameter combinations to control land use changes, and then translating the parameter combination that can reflect the effect of land use policies into the policy effectiveness. This process is realized by ArcGIS and GeoSOS-FLUS software developed based on Cellular Automata (CA) model. The method was successfully tested in the peri-urban region near Shanghai metropolitan area. The raw material is Land-Use/Cover (LULC) data of study area in 2000 and 2015. This method assessed the effects of land use policies during fifteen years, and analyzed the mechanism of effective policies, as well as the types and reasons of failure policies. This presented method is useful in land governance and the formulation and implementation of land use policy.

• This article developed a backcasting approach to evaluate the effectiveness of land use policies on land use changes in a certain period of time.
• The policy action mechanism was transformed into controllable, visible, and adjustable parameter combinations.
• The method built an analytical framework for the assessment of land use policy effectiveness in any regions.

## Graphical abstract

**Figure:**
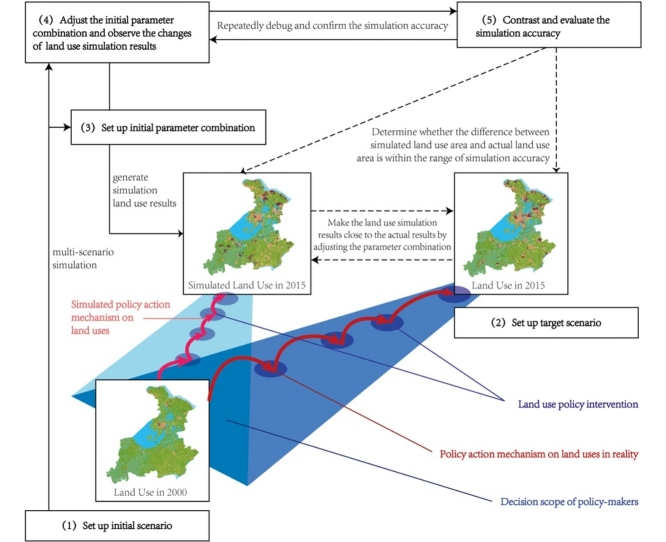

**Specifications Table**

**Table:** 

Subject Area:	Environmental Science
More specific subject area:	Land use, landscape changes, land use policy
Method name:	Backcasting approach with multi-scenario simulation (BAMSS)
Name and reference of original method:	Dreborg, K. H., (1996). Essence of backcasting. Futures, 28(9), 813-828. Holmberg, J., & Robert, K. H., (2000). Backcasting — a framework for strategic planning. International Journal of Sustainable Development & World Ecology, 7(4), 291-308.
Resource availability:	Website of GeoSOS-FLUS software: http://www.geosimulation.cn/index.html

## Method details

### Background

Land use regulations, policies and laws are important for governments to allocate land resources, make executive decisions, and coordinate the relationships among stakeholders, so that mutual interests of social development units are realized [[1], [2], [3]]. Land use is influenced by urban development and controlled by land use policy [4]. Under the pressure of rapid urban sprawl, land use policies have been formulated to conserve cultivated land and natural spaces. But the changes in the quantity and scale of cultivated land and natural spaces are still significant, and most of them are reduced in area. Therefore, we speculate that there is policy failure or abuse behind these changes. However, we are not clear about the influencing mechanism and actual operation effect of land use policy on land use changes. Therefore, it is necessary to assess the effects of land use policies to explain and regulate land use changes. Achieving this goal is difficult, because Land-Use/Cover Changes (LUCC) are affected by a variety of land use policies, such as land management policies [5], green space conservation policies [6], planning and management policies [7] and fiscal incentive policies [8], and the effects of these policies promote or offset each other.

**Fig. 1:**
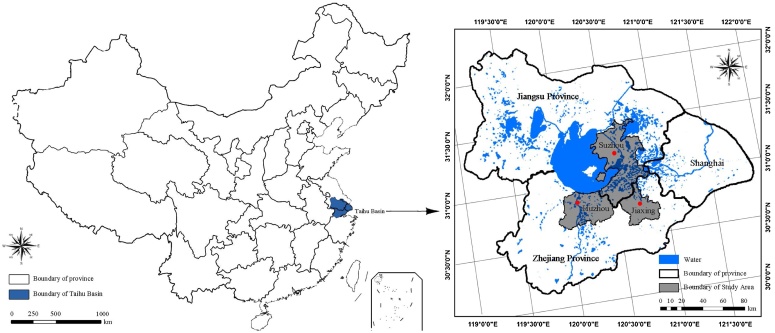
The map of the study area, the information includes the administrative boundaries of Suzhou, Jiaxing, Huzhou and Shanghai, the drainage system of Taihu lake basin and the provincial administrative boundaries of Jiangsu province and Zhejiang province.

In this study, a backcasting approach is used [9]. Different from other methods of multi-scenario simulation based on current situation to predict futures, this multi-scenario simulation method of land use based on backcasting approach focuses on exploring possible causes and solutions to problems existing in a certain period of time under the influence of multiple factors such as policy. It enables the separation of land use policy factor to analyze its direct impact on land use changes over time. In this study, GeoSOS-FLUS software developed based on Cellular Automata (CA) model was used as a tool to simulate multiple land use change scenarios. Based on the simulation of LUCC during 2000–2015 in Suzhou, Jiaxing and Huzhou areas (Su-Jia-Hu Area, for short) which are in peri-urban region near Shanghai (Fig. 1), the impact of development and the implementation of land use policies on land uses are discussed, and a basis for improving and implementing land use policies is provided.

### Data used

The remote sensing image data used in this study is from Landsat satellites, downloading from the USGS (http://glovis.usgs.gov/) website. The original image was synthesized in true color, and the final image result was formed through fusion, mosaic and other processing, with a resolution of 15 m. All the land use raster data of Su-Jia-Hu Area in 2000 and 2015 used in the study were artificially visualized, manually sketched and vectored, and then converted into raster data. With reference to the national standard "Land use Present Situation Classification" (GB/T 21010-2017) revised by the Ministry of Land and Resources of the People's Republic of China, and in combination with the characteristics of land use in Su-Jia-Hu Area and the decidability of remote sensing information, the land use in the study area is divided into twelve types: forest, pasture, river, lake and pond, wetland, unused land, cultivated land, city and town areas, rural residential land, land for transport, industrial land and other construction land.

### Analytical framework for the multi-scenario simulation of land use changes

**Fig. 2:**
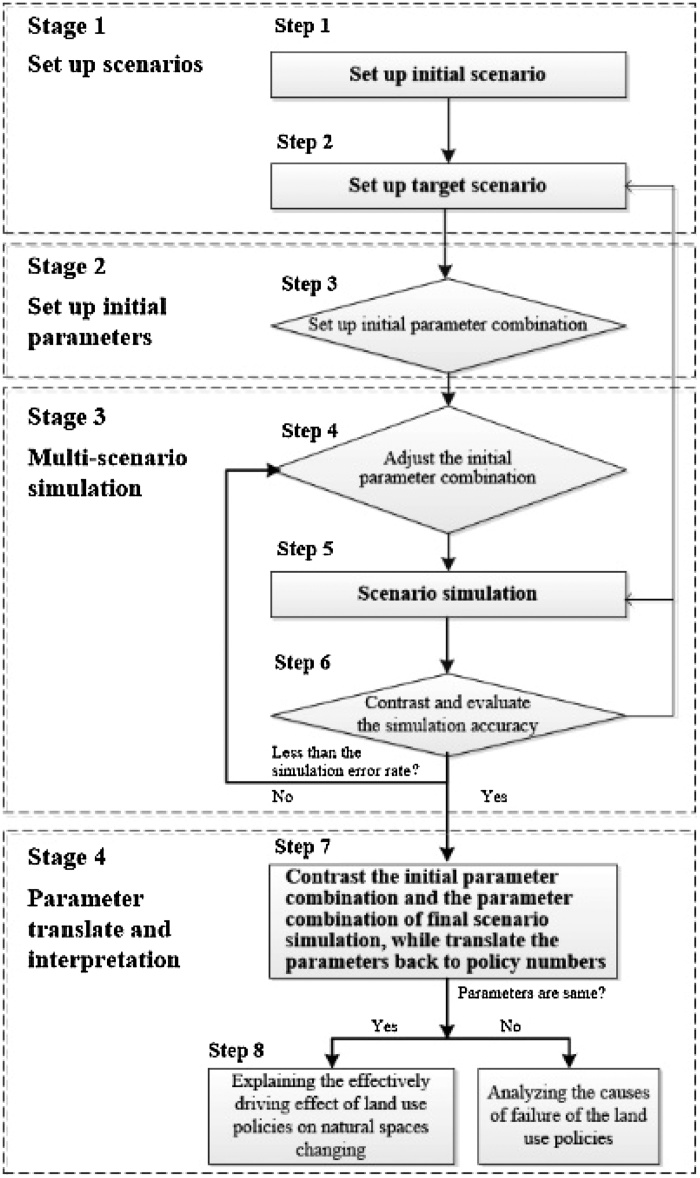
The analytical framework for the multi-scenario simulation of land use changes to assess the effects of land use policy, including the process of expressing the policy action mechanism as the parameter combinations to control land use changes, and then translating the parameter combination that can reflect the effect of land use policies into the policy effectiveness.

The method is structured in four sequential stages, including the process of expressing the policy action mechanism as the parameter combinations to control land use changes, and then, through the screening of the results of multi-scenario simulation, translating the selected parameter combination that can reflect the effect of land use policies into the policy effectiveness. The analytical framework for multi-scenario simulation of land use changes is shown in Fig. 2.

### Stage 1 in detail: set up initial and target scenario (Steps 1–2)

Determining the initial and target scenario is the first stage of the multi-scenario simulation based on backcasting approach. In this study, the initial scenario is the land use of Su-Jia-Hu Area in the year of 2000, the target scenario is the land use of Su-Jia-Hu Area in the year of 2015. By using the property table of ArcGIS, the area data of each type of land use in 2000 and 2015 were calculated and sorted into Table 1.

**Table 1:** The land use area of Su-Jia-Hu Area in the year of 2000 and 2015.

Land use type		Area in 2000 (km^2^)	Area in 2015 (km^2^)
Natural spaces	Forest	2750.79	2695.16
	Pasture	101.03	100.82
	River	633.68	608.71
	Lake and pond	2737.29	3042.88
	Wetland	99.33	101.36
	Unused land	7.44	12.69
Subtotal		**6329.56**	**6561.62**
Unnatural spaces	Cultivated land	10112.79	7705.93
	City and town areas	572.10	1603.23
	Rural residential land	1227.68	1458.70
	Land for transport	92.84	233.91
	Industrial land	145.04	778.12
	Other construction land	55.90	194.40
Subtotal		**12206.35**	**11974.28**
Total		**18535.91**	**18535.91**

### Stage 2 in detail: set up initial parameter combination (Step 3)

The initial parameter combination represents the specific constraints or action mechanisms during the transformation from the initial scenario to the target scenario. In this study, the initial parameter combination is set as cost parameters in the conversion cost matrix according to the comprehensive effect of land use related regulations, policies, laws and documents during 2000–2015. The specific steps to obtain the initial parameter combination are as follows:

(1) **Land use policy collection.** Considering the delays from the promulgation to implementation of the policy, we searched the policies of the three cities and their subordinate provinces from 1995 to 2015. Through the interpretation of the purpose of the promulgation and the action lever, we screened 46 related policies that have direct or indirect influence on land use changes of Su-Jia-Hu Area. Among them, 37 policies apply to the conversion between natural spaces and unnatural spaces, 4 policies apply to conversion within natural spaces, and 16 policies apply to conversion within unnatural spaces; some policies have various effects on land use changes (see Table S1 in Supplementary material). Information sources were the official websites of the People's Governments of the three cities of Su-Jia-Hu Area and of Jiangsu and Zhejiang provinces, the website of the environmental conservation bureau, the website of the land and resources bureau, and the bulletin of the People's Government.
(2) **Classification of land use policies.** According to the research purpose, firstly, divide the land use in the study area into two categories, natural spaces and unnatural spaces. The land use of natural spaces include: forest, pasture, river, lake and pond, wetland and unused land. The types of unnatural spaces include: cultivated land, city and town areas, rural residential land, land for transport, industrial land and other construction land. Then land use policies are divided into three types according to the land conversion among the categories: conversion between natural spaces and unnatural spaces; conversion within natural spaces; and conversion within unnatural spaces, which mainly refers to conversion between construction land and cultivated land.
(3) **Land use policy effect intensity grading and assignment.** The policies that directly affect the land use are classified as "Direct influence" and denoted as "D (Direct)". Policies that act on other levels of the region, but ultimately promotes or inhibits the conversion of land use is classified as "Indirect influence" and denoted as "I (Indirect)". Mandatory policies, such as those with strong legal effect in the process of land use conversion, and those with mandatory language, such as "strict", "prohibited" and "must" (that is, mandatory conversion or mandatory prohibition of conversion), are classified as "Strong influence" and denoted as “S (Strong)”. Other land use policies are classified as “Weak influence” and denoted as “W (Weak)”. The action direction of land use policy is divided into "Positive (+)" and "Negative (−)". The former represents a beneficial effect on natural spaces, including "the incentive to convert to natural spaces with higher ecosystem services" and "the restriction to transfer from natural spaces with higher ecosystem services", while the latter is the opposite. The policy assignment Q of different intensity for land use changes can be expressed as:

(1) Q = PM

(2) $$\text{Where}:\text{P}=\;\left\{\begin{array}{l} 9,\;if\;the\;intensity\;is\;D-S(Directly\;strong\;impact)\\ 5,\;if\;the\;intensity\;is\;D-W\;or\;I-S(Directly\;weak/indirectly\;strong\;impact)\;\\ 1,\;if\;the\;intensity\;is\;I-W(Indirectly\;weak\;impact) \end{array}\right)$$

(3) $$\text{M}=\left\{\begin{array}{c} 1,\;if\;the\;direction\;is\;“+”\\ -1\;if\;the\;direction\;is\;“-” \end{array}\right)$$

The 46 land use policies collected were numbered according to "policy number - effect intensity - effect direction" to form the list of land use policy action mechanism number and assignment (see Table S1 in Supplementary material).

(4) **Land use conversion cost matrix** (see Table S2 in Supplementary material). Land use policy corresponds with land use changes caused by the policy, and the comprehensive mechanism of land use policy is represented by the sum of policy assignment. The assignment V of the parameters of each cell in the land use conversion cost matrix can be expressed as:

(4) $$\begin{array}{c} \text{Value}=\frac{\sum_{k\in n_{ij}}Q_{k}}{max\left\{\sum_{k\in n_{ij}}Q_{k}\right\}}\\ \text{if}\;\text{min}\left\{Value\right\}\geq 0,V=Value\;;\\ \text{if}\;\text{min}\left\{Value\right\}<0,V=Value+\left|min\left\{Value\right\}\right| \end{array}$$

where, i represents the type of land use transferred out; j represents the type of land use transferred in; $n_{ij}$ denotes a set of policy numbers for converting i-type land use into j-type land use; k denotes the policy number, and 1≤k≤52  (because of the multifaceted nature of the policy action mechanism, the number of the policy action mechanisms was increased to 52, see Table S1 in Supplementary material); $Q_{k}$ denotes the corresponding assignment of the policy number, as shown in Table S1. Where, " V = 0″ stands for "prohibition of conversion/non-conversion", "  V = 1″ stands for "complete conversion/encouragement of conversion", and "  V $\in \left(0,1\right)$" stands for different land conversion costs from low to high. This conversion cost matrix is the initial parameter of multi-scenario simulation of land use changes in Su-Jia-Hu Area from 2000 to 2015.

(5) **Land use expansion coefficient** (see Table S3 in Supplementary material). This parameter is used to set the neighborhood factor parameters of the various land types. The land use expansion capacity of Su-Jia-Hu Area is closely related to the development stage and main growth point of the area from 2000 to 2015. The land use expansion coefficient $W_{u}$ in Su-Jia-Hu Area can be expressed as:

(5) $$W_{u}=\frac{R_{u}}{max\left\{R_{u}\right\}}$$

(6) $$\begin{array}{c} \text{Where}:Rate_{u}=\frac{A_{u2015}-A_{u2000}}{A_{u2000}\times 15}\times 100\%\;\\ \text{if}\;\text{min}\left\{Rate_{u}\right\}\geq 0,R_{u}=Rate_{u};\\ \text{if}\;\text{min}\left\{Rate_{u}\right\}<0,R_{u}=Rate_{u}+\left|min\left\{Rate_{u}\right\}\right| \end{array}$$

where, u denotes a land use type; $R_{u}$ denotes the average annual growth rate of u -type land between 2000 and 2015; $A_{u2015}$ denotes the area of u -type land in 2015; $A_{u2000}$ denotes the area of u -type land in 2000.

### Stage 3 in detail: adjust the initial parameter combination to get the parameter combination of final scenario simulation (Steps 4–6)

**Fig. 3:**
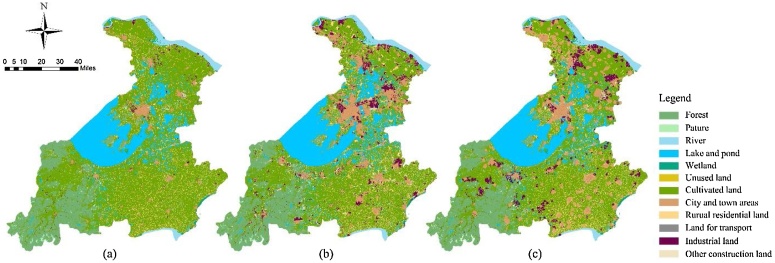
Land use status and simulation results of Su-Jia-Hu Area from 2000 to 2015, (a) is the land use status map in 2000; (b) is the land use status map in 2015; (c) is the land-use simulation map of 2015 under initial parameter combination.

This stage requires repeated cycles of steps to achieve the goal. In this stage, the initial parameter combination obtained in the previous stage is used as the initial conversion cost matrix input in the GeoSOS-FLUS software, and the initial simulation scenario is generated (Fig. 3). The area statistics of each land use type in the simulation were collected and compared with the actual land use situation in 2015, and the systematic error (the ratio of the difference between the simulated land use area in 2015 and the land use area in 2010 to the latter) was calculated (Table 2). The initial simulation scenario represents the land use situation in Su-Jia-Hu Area in 2015 under an ideal situation where all policies have been acting according to the effects theoretically analyzed as shown in Table S1. However, in fact, According to the area statistics in Table 2, there is an error between the simulated land use scenario and the actual scenario in 2015 under the action of the initial parameter combination determined by the policy. This indicates that the land use policy represented by the initial parameter combination is not completely consistent with the actual situation of land use changes in 2000–2015 in Su-Jia-Hu Area under the action of the policies. The two columns of "Absolute error" and "Systematic error" in Table 2 respectively indicate the difference and proportion of errors of various types of land use.

**Table 2:** Comparison of scenario simulation results under initial parameter combination.

Land use type		Actual land area in 2000 (km^2^)	Actual land area in 2015 (km^2^)	Simulation land area in 2015 (km^2^)	Absolute error (km^2^)	Systematic error (%)
Natural spaces	Forest	2750.79	2695.16	2740.94	45.78	**1.70**
	Pasture	101.03	100.82	101.03	0.21	**0.20**
	River	633.68	608.71	633.68	24.98	**4.10**
	Lake and pond	2737.29	3042.88	2721.23	−321.65	**−10.57**
	Wetland	99.33	101.36	98.65	−2.71	**−2.67**
	Unused land	7.44	12.69	6.62	−6.07	**−47.80**
Unnatural spaces	Cultivated land	10112.79	7705.93	8246.12	540.18	**7.01**
	City and town areas	572.10	1603.23	1603.23	0.00	0.00
	Rural residential land	1227.68	1458.70	1458.70	0.00	0.00
	Land for transport	92.84	233.91	92.84	−141.06	**−60.31**
	Industrial land	145.04	778.12	778.12	0.00	0.00
	Other construction land	55.90	194.40	54.75	−139.65	**−71.84**

The systematic error is the ratio of the difference between the simulated land use area in 2015 and the land use area in 2010 to the latter. System errors are used to represent the accuracy of the simulation. The system error within ±5% conforms to the accuracy range.

Taking into account the total sample data, the allowable error of the systematic error of land use area was set as ±5% which is the accuracy range of the total land use area as well as the single type of land use area. As can be seen from Table 2, the differences between the simulated value and the actual value of the area of several land use types fall outside the accuracy range. Therefore, it is necessary to adjust the parameters based on the initial simulation parameter combination. By constantly adjusting the initial parameter combination and observing how different parameter combinations change the land use, the land use area of the simulation scenario is finally close to the target scenario in reality. The adjustment rules for initial parameter combination within the conversion cost matrix are: (1) The land use type is converted from A to B. If the simulation result of A is higher than the actual one and the simulation result of B is lower than the actual one, it indicates that the cost parameter setting of the conversion from A to B is too low and needs to be appropriately improved; (2) If the simulation result of A is lower than the actual result and that of B is higher than the actual result, it indicates that the cost parameter setting of the conversion from A to B is too high and needs to be reduced appropriately; (3) Other circumstances will not be adjusted for the time being. Specific adjustment of the parameters is based on the practical experience of land use conversion and the conversion rules set by the initial parameter combination. In that situation, as far as possible close to the target scenario at the same time, the less is as far as possible on each parameter adjustment and adjust the number of less as far as possible, to ensure that simulation parameters have practical value and policy meaning. Keeping the simulated scenario as close as possible to the target scenario, the adjustment range and adjustment times of each parameter should be as small as possible to ensure that the simulation scenario parameters have realistic value and policy significance.

**Fig. 4:**
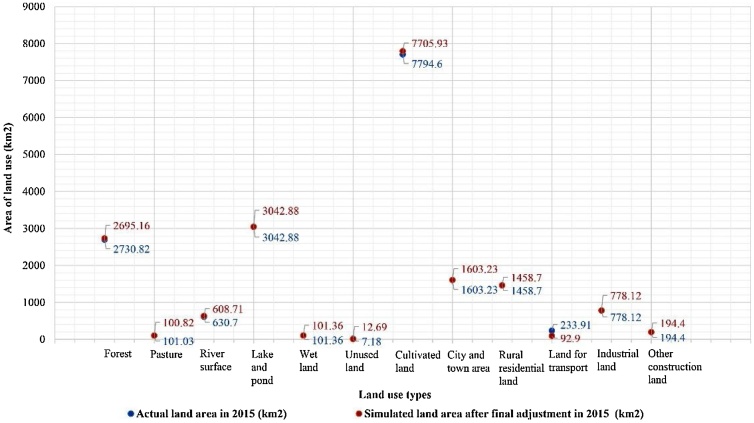
A scatter plot shows the difference between the simulated results and the actual results of various land use areas after parameter adjustment in 2015.

**Fig. 5:**
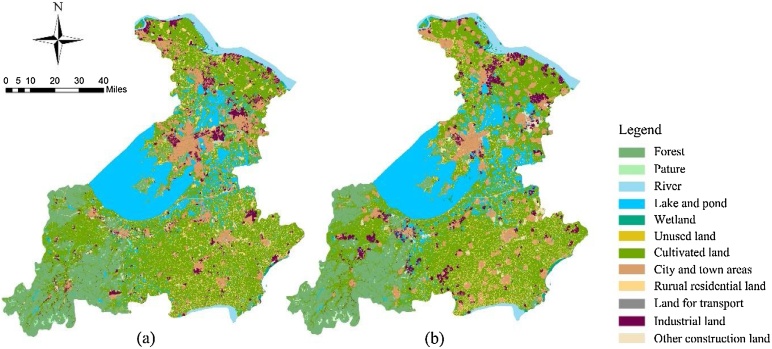
Land-use status, simulation results and simulation adjustment results of Su-Jia-Hu Area of 2015, (a) is the land use status map in 2015; (b) is the land use simulation map after parameter adjustments of 2015.

According to the above parameter adjustment rules and the initial simulation results, the initial parameter combination was adjusted, and the land use scenario simulation results were generated. Then the simulation results were compared with the actual results, and whether to continue parameter adjustment depends on whether the simulation accuracy is within the simulation error range. In this study, a total of five rounds of parameter combinations were adjusted and simulated, and the errors between the simulated land use areas and the actual results were finally within the accuracy range. According to the scatterplot in Fig. 4, the gap between the simulation results and the actual results can be seen more clearly. The conversion cost matrix obtained by parameter adjustments and the statistical table of land use scenario simulation results generated under each matrix were given in the Section of Additional Information. The specific simulation results after adjustment are shown in Fig. 5, the statistics of various land use areas are shown in Table 3, and the parameter combination of the final simulation scenario as conversion cost matrix parameters is shown in Table S4 in Supplementary material. The parameter combination of final scenario simulation refers to the parameters that make the scenario simulation results basically consistent with the target scenario. To some extent, they can reflect the real effect of the policy.

**Table 3:** Comparison of results under the parameter combination of final scenario simulation.

Land use type		Actual land area in 2015 (km^2^)	Simulated land area after final adjustment in 2015 (km^2^)	Absolute error (km^2^)	Systematic error (%)
Natural spaces	Forest	2695.16	2730.82	35.66	1.31
	Pasture	100.82	101.03	0.21	0.20
	River	608.71	630.70	21.99	3.61
	Lake and pond	3042.88	3042.88	0.00	0.00
	Wetland	101.36	101.36	0.00	0.00
	Unused land	12.69	7.18	−5.51	**−43.41**
Unnatural spaces	Cultivated land	7705.93	7794.60	88.67	0.47
	City and town areas	1603.23	1603.23	0.00	0.00
	Rural residential land	1458.70	1458.70	0.00	0.00
	Land for transport	233.91	92.90	−141.00	**−60.28**
	Industrial land	778.12	778.12	0.00	0.00
	Other construction land	194.40	194.40	0.00	0.00

The systematic error of "Unused land" is still relatively large (−43.41%) after the adjustments, however, considering its total amount is small (12.69 km^2^), and its absolute error (5.51 km^2^) is far less than that of other land use types, thus the influence of a large systematic error of "Unused land" on the adjusted simulation results can be ignored. CA model is mainly applicable to the simulation of block land space, but can’t accurately simulate the change of linear space, such as land for transport. In addition, the actual area of transport land (233.91 km^2^) in 2015 accounts for a small proportion (1.26%) of the total land area, so no parameter adjustments for the relatively large error (60.28%) of land for transport will not affect the conclusion of this study. In addition to "Unutilized land" and "Land for transport", the systematic errors of various land use types are all controlled within ±5%, which generally agrees with the accuracy range of the simulation evaluation.

### Stage 4 in detail: translate the parameter combination and analyze its invalidation (Step 7–8)

This stage is the last two steps of applying this method to assess the effects of land use policies. Firstly, contrast the initial parameter combination and the parameter combination of final scenario simulation in Tables S2 and S4. For the same part of the parameters that can reflect the evolution characteristics and rules of land-use/cover in this period under specific constraint conditions or action mechanisms, translate them back into policy numbers and explain the effectiveness of land use policies on natural spaces changing. For the different part of the parameters which show the ineffectiveness of land-use/cover mechanism, after translating back to the policies they represent, it is necessary to analyze possible failure causes of these land use policies. The conversion cost parameters in Tables S2 and S4 are translated into the land use policy numbers as follows: first, the values in each cell in the initial conversion cost matrix (Table S2) are reduced to the policy number set for converting i -type land to j -type land, namely $n_{ij}$ in formula (4). Secondly, the $n_{ij}$ in each cell is corresponding to the parameter adjustment type in the scenario simulation conversion cost matrix (Table S4). The $n_{ij}$ corresponding to the class "Up regulation" or "Down regulation" represents the invalidation policy number set, and the $n_{ij}$ corresponding to the class "Without changes" represents the effective policy number set. After translating the parameter values into the corresponding policy action mechanism numbers (“policy number” and “policy intensity and direction”), the land use policies are classified and graded (see Tables S5 and S6 in Supplementary material), which reflect the effect and intensity of the land use policies on land use changes in Su-Jia-Hu Area from 2000 to 2015. Compared with the initial parameter combination, the policies corresponding to the simulation parameters after adjustments are defined as "failure policy"; the policies corresponding to the simulation parameters without adjustments, which are consistent with the initial parameter combination are defined as "effective policy". "Effective policy" refers to a policy that makes the land use simulation results closer to the actual results in the total amount of land used and the spatial structure under the condition of no adjustment. "Failure policy" can be divided into "spatial structure failure policy" and "land use total quantity failure policy". "Spatial structure failure policy" means that although the land use area is within the accuracy range (±5%) under the adjusted parameters, the spatial distribution is significantly different from the actual results. "Land use total quantity failure policy" means that the spatial distribution of simulation results is basically consistent with the actual results, but the total quantity of the land use area isn’t within the accuracy range (±5%), before the initial parameter combination adjustment. For effective policies, they can be divided into four categories according to the land use type of the policy function: Category A, direct natural spaces protection policies; Category B, non-mandatory policies to restrict and manage the potential conversion of natural spaces into unnatural spaces; Category C, policies that provide a legal basis for the conversion of natural spaces into unnatural spaces; Category D, direct cultivated land protection policies. The focus of the analysis of the mechanism of the above effective policies is to find the leverage of the policies so as to find the reasons for promoting the conversion from one type of land use to another. As for the failure policy, firstly, the failure reasons of "spatial structure failure policy" are analyzed qualitatively. Secondly, the failure degree of "land use total quantity failure policy" is classified according to the range of parameter adjustment: policies with low failure degree, medium failure policy and high failure degree policy. The "inefficient policy" affects land use conversion results less than the simulation results; the "excessive policy" affects land use conversion results more than the simulation results; the "absent policy" means that land use conversion is carried out in the absence of corresponding land use policy control. Then, combining the types of land use with the role of failure policy, the possible failure causes of inefficient policies, excessive policies and absent policies were analyzed.

Based on the analysis results, the list of policies that have played a role in land use changes between 2000 and 2015 in Su-Jia-Hu Area was finally obtained (see Table S5 in Supplementary material). At the same time, the list of failures policies during this period, the types and degrees of the failure policies, as well as the impact of policies’ ineffectiveness on various types of land use were obtained (see Table S6 in Supplementary material), and the reasons behind policy failures were speculated and analyzed, which will serve as an important basis for adjusting the formulation of land use policies and strengthening the implementation of them. This proves that the use of GeoSOS-FLUS software and the multi-scenario simulation of land use change based on the backcasting approach can become a method to assess the effects of policies on land use changes.
