# Supplementary material for: Backcasting approach with multi-scenario simulation for assessing effects of land use policy using GeoSOS-FLUS software
Source: MethodsX. 2019 May 17;6:1384–97. doi: 10.1016/j.mex.2019.05.007 (PMC6579905; doi:10.1016/j.mex.2019.05.007)
Supplement: Supplementary file 1 [file mmc1.docx]

**Supplementary material**

We list the tables that show the processing and analysis of the data here. The table numbers correspond to the annotations in the main text.

**Table S1.**

Classification, numbered and assignment list of land use policies.

| **No.** | **Classification** | **Policy name** | | **Policy action mechanism number** | | **Policy assignment** |
| --- | --- | --- | --- | --- | --- | --- |
|  |  |  |  | **Policy number** | **Policy intensity and direction** |  |
| 01 | N-UN | *Notice on strengthening wetland conservation and management by the general office of Jiangsu provincial people's government (2004)* | | 01- | D-S-+ | 9 |
| 02 |  | *Lake conservation regulation of Jiangsu province (2004)* | | 02- | D-S-+ | 9 |
| 03 |  | *Planning of ecosystem and environment functional zone of Wuxing district, Huzhou city (2007)* | | 03- | D-S-+ | 9 |
| 04 |  | *Planning of Jiaxing ecological functional zone (2008)* | | 04- | D-S-+ | 9 |
| 05 |  | *Planning of ecological civilization construction in Jiaxing city (2014)* | | 05- | D-S-+ | 9 |
| 06 |  | *Opinions on further strengthening lake management and conservation (2010) (Suzhou)* | | 06- | D-S-+ | 9 |
| 07 |  | *Suzhou eco-civilization construction plan (2010 ~ 2020)* | | 07- | D-S-+ | 9 |
| 08 |  | *Environmental conservation plan for Suzhou city during the 11th five-year plan period (2006-2010)* | | 08- | D-S-+ | 9 |
| 09 |  | *Environmental conservation plan for Suzhou city during the 12th five-year plan period (2011-2015)* | | 09- | D-S-+ | 9 |
| 10 |  | *Implementation opinions of Suzhou main functional area (2014)* | | 10- | D-S-+ | 9 |
| 11 |  | *Planning of Huzhou ecosystem and environment functional zone (2015)* | | 11- | D-S-+ | 9 |
| 12 |  | *Division scheme of water environment functional area in Zhejiang province (2015)* | | 12- | D-S-+ | 9 |
| 13 | N-N/ UN-UN | *Land management regulations of Jiangsu province (2000)* | | 13- | D-W-+ | 5 |
|  |  |  |  | 14- | D-S-+ | 9 |
| 14 | UN-UN | *Emergency notice of the general office of the state council on in-depth management and rectification of the land market and strict land management (2004)（Zhejiang Province）* | | 15- | D-S-+ | 9 |
| 15 |  | *Notice on the establishment of provincial basic farmland conservation demonstration area (2006) (Jiangsu province)* | | 16- | D-S-+ | 9 |
| 16 | N-UN | *Administrative measures for the development and utilization of mudflats in Jiangsu province (1998)* | | 17- | D-W- - | -5 |
| 17 | N-UN/ N-N | *Forest management regulations of Zhejiang province (2004)* | | 18- | D-W-+ | 5 |
| 18 | N-UN | *Notice of Zhejiang provincial people's government on strict land management and effective improvement of land use efficiency (2004)* | | 19- | D-W- - | -5 |
|  | UN-UN |  |  | 20- | D-W-+ | 5 |
| 19 | N-UN | *Notice of Zhejiang provincial people's government on scientific development and utilization of mudflats resources (2005)* | | 21- | D-W- - | -5 |
| 20 |  | *Forest land management measures of Zhejiang province (2005)* | | 22- | D-W- - | -5 |
| 21 |  | *Opinions of Zhejiang provincial people's government on the implementation of the state council's decision on deepening reform and strict land management (2005)* | | 23- | D-W- - | -5 |
| 22 | N-UN/ N-N | *Opinions on strengthening environmental remediation of abandoned open-pit mining mountains (2005) (Jiangsu province)* | | 24- | D-W- - | -5 |
|  |  |  |  | 25- | D-W-+ | 5 |
| 23 | N-UN | *Opinions of Suzhou municipal people's government on implementing the decision of the state council to deepen reform and strictly manage land (2005)* | | 26- | D-W- - | -5 |
|  | UN-UN |  |  | 27- | D-W-+ | 5 |
| 24 | N-UN | *Notice of Huzhou municipal people's government on printing and distributing the interim measures for rural land development in Huzhou city (2005)* | | 28- | D-W- - | -5 |
| 25 |  | *Regulations on public welfare forests in Jiangsu province (2006)* | | 29- | D-W-+ | 5 |
| 26 |  | *Notice on further strengthening land development and consolidation (2007) (Jiangsu province)* | | 30- | D-W- - | -5 |
| 27 |  | *Notice of Zhejiang provincial department of land and resources on the implementation of the target of mudflats reclamation as a whole and supplementary cultivated land (2007)* | | 31- | D-W- - | -5 |
| 28 | N-UN/ UN-UN | *Several opinions on integrated development of urban and rural areas in Suzhou (2008)* | | 32- | D-W-+ | 5 |
|  |  |  |  | 33- | D-W- - | -5 |
| 29 | N-UN | *Suzhou national ecological city construction work plan (2008)* | | 34- | D-W-+ | 5 |
| 30 |  | *Annual work plan for comprehensive improvement of rural environment in Suzhou (2010)* | | 35- | D-W-+ | 5 |
| 31 | N-UN/ UN-UN | *Regulations on land remediation in Zhejiang province (2014)* | | 36- | D-W-+ | 5 |
|  |  |  |  | 37- | D-W- - | -5 |
| 32 | N-UN/ N-N | *Punishment for abandoning cultivated land in Zhejiang province (1998)* | | 38- | I-S-+ | 5 |
| 33 | N-UN/ UN-UN | *Provisional regulations on administrative punishment of illegal land management in Jiangsu province (1999)* | | 39- | I-S-+ | 5 |
| 34 | UN-UN | *Notice of Zhejiang provincial people's government on administrative measures for land reclamation fees collection (2001)* | | 40- | I-S-+ | 5 |
| 35 |  | *Emergency notice of Jiangsu provincial department of land and resources on further strengthening of land law enforcement and supervision to strictly investigate and deal with land violations (2004)* | | 41- | I-S-+ | 5 |
| 36 |  | *Opinions on the implementation of the pilot program of comprehensive supporting reform in urban and rural areas (2008) (Jiaxing)* | | 42- | I-S-+ | 5 |
| 37 | N-UN | *Rules for the implementation of Suzhou eco-compensation regulations (2014)* | | 43- | I-S-+ | 5 |
| 38 | UN-UN | *Methods of diversion of land requisitioning personnel in urban areas of Jiaxing (1998)* | | 44- | I-W- - | -1 |
| 39 | N-UN | *Overall planning for soil and water conservation in Zhejiang province (2001)* | | 45- | I-W-+ | 1 |
| 40 |  | *Notice of Huzhou municipal people's government on the issuance of interim measures for the management of urban land reserve in Huzhou (2002)* | | 46- | I-W- - | -1 |
| 41 | N-UN /UN-UN | *Outline of Zhejiang ecological province construction plan (2003)* | | 47- | I-W-+ | 1 |
| 42 |  | *Pilot measures for compensation for land expropriation and basic living security for peasants expropriated in Suzhou (2004)* | | 48- | I-W- - | -1 |
| 43 | N-UN | *Notice on exemption of agricultural tax in Jiaxing city (2004)* | | 49- | I-W- - | -1 |
| 44 |  | *Several opinions of Huzhou people's government on strict land management to promote intensive land use (2005)* | | 50- | I-W- - | -1 |
| 45 | UN-UN | *Interim measures for the administration of rural collective land expropriation in Jiaxing city (2009)* | | 51- | I-W- - | -1 |
| 46 |  | *Measures for the basic living security of land-expropriated farmers in Zhejiang province (2010)* | | 52- | I-W- - | -1 |
|  |  | | | | | |
| N-UN | |  | The transition between natural space and unnatural space. | | | |
| N-N | |  | The transition within natural spaces. | | | |
| UN-UN | |  | The transition within unnatural spaces. | | | |

**Table S2.**

Initial parameter combination - Land use conversion cost matrix driven by policy.

**Table S3.**

The growth rate and land expansion coefficient of land use in 2000-2015.

| **Land use type** | | **Increased area (km^2^)** | **Annual rate of growth (%)** | **Land expansion coefficient treated by normalization** |
| --- | --- | --- | --- | --- |
| **Natural spaces** | **Forest** | -55.63 | -0.13 | 0.04 |
|  | **Pasture** | -0.21 | -0.01 | 0.05 |
|  | **River** | -24.98 | -0.26 | 0.04 |
|  | **Lake and pond** | 305.60 | 0.74 | 0.08 |
|  | **Wetland** | 2.03 | 0.14 | 0.06 |
|  | **Unused land** | 5.24 | 4.70 | 0.21 |
| **Subtotal** | | **232.06** | **—** | **—** |
| **Unnatural spaces** | **Cultivated land** | -2406.86 | -1.55 | 0.01 |
|  | **City and town areas** | 1031.13 | 12.02 | 0.45 |
|  | **Rural residential land** | 231.02 | 1.25 | 0.09 |
|  | **Land for transport** | 141.07 | 10.13 | 0.38 |
|  | **Industrial land** | 633.08 | 29.10 | 1.00 |
|  | **Other construction land** | 138.49 | 16.52 | 0.59 |
| **Subtotal** | | **-232.06** | **—** | **—** |
| **Total** | | **0** | **—** | **—** |

**Table S4.**

The parameter combination of final scenario simulation - Land use conversion cost matrix driven by policy.

**Table S5.**

Summary of effective policy action mechanism numbers and mechanism.

| **Type** | **Policy action mechanism numbers** | | | | | **Policy mechanism** | |
| --- | --- | --- | --- | --- | --- | --- | --- |
| **Effective policy** | 04-D-S-+  10-D-S-+ | 05-D-S-+  11-D-S-+ | 07-D-S-+  29-D-W-+ | 08-D-S-+ | 09-D-S-+ | Forest conservation | **Category A** |
|  | 03-D-S-+  09-D-S-+ | 04-D-S-+  10-D-S-+ | 05-D-S-+  11-D-S-+ | 07-D-S-+  12-D-S-+ | 08-D-S-+ | River conservation |  |
|  | 03-D-S-+  08-D-S-+ | 04-D-S-+  09-D-S-+ | 05-D-S-+  10-D-S-+ | 06-D-S-+  11-D-S-+ | 07-D-S-+  12-D-S-+ | Lake and pond conservation |  |
|  | 04-D-S-+  10-D-S-+ | 05-D-S-+  11-D-S-+ | 07-D-S-+ | 08-D-S-+ | 09-D-S-+ | Wetland conservation |  |
|  | 35-D-W-+ | 45-I-W- + |  |  | | Cultivated land→ Forest | **Category B** |
|  | 32-D-W-+ | | | | | City and town areas→ Forest |  |
|  | 34-D-W-+ | 35-D-W-+ | | | | Rural residential land→ Forest |  |
|  | 32-D-W-+ | | | | | Other construction land→ Forest |  |
|  | 32-D-W-+ | | | | | City and town areas→ Wetland |  |
|  | 32-D-W-+ | | | | | Other construction land→ Wetland |  |
|  | 37-D-W- - | 43-I-S-+ |  | | | Forest→ Cultivate land | **Category C** |
|  | 22-D-W- - | 48-I-W- - |  | | | Forest→ City and town areas |  |
|  | 02-D-S-+ | 48-I-W- - | | | | Lake and pond→ City and town areas |  |
|  | 01-D-S-+ | 17-D-W- - | 43-I-S-+ | | | Wetland→ City and town areas |  |
|  | 26-D-W- - | 30-D-W- - | 46-I-W- - | 48-I-W- - | | Unused land→ City and town areas |  |
|  | 17-D-W- - | 21-D-W- - | | | | Wetland→ Land for transport |  |
|  | 46-I-W- - | | | | | Unused land→ Land for transport |  |
|  | 17-D-W- - | 21-D-W- - | 43-I-S- + | | | Wetland→ Other construction land |  |
|  | 14-D-S-+  48-I-W- - | 20-D-W-+  50-I-W- - | 40-I-S-+  51-I-W- - | 41-I-S-+  52-I-W- - | 44-I-W- - | Cultivated land→ City and town areas |  |
|  | 40-I-S-+ | 52-I-W- - | | | | Cultivated land→ Rural residential land |  |
|  | 15-D-S-+ | 16-D-S-+ | 34-D-W-+ | 43-I-S-+ | 47-I-W-+ | Cultivated land conservation | **Category D** |
|  | 22-D-W- - | 27-D-W-+ | 36-D-W-+ | 42-I-S-+ | | Rural residential land→ Cultivated land |  |

**Table S6.**

Summary of failure policy action mechanism numbers, degree of failure and mechanism.

| **Type** | | **Degree of failure** | **Policy action mechanism numbers** | **Policy mechanism** | **The simulation results compared to actual results** | **Types of actual policy failures** |
| --- | --- | --- | --- | --- | --- | --- |
| **Failure policy** | **Spatial structure failure policy** | — | 24-D-W- - 26-D-W- - | Unused land→ Industrial land | — | — |
|  |  |  | 33-D-W- - | Cultivated land→ Industrial land | — | — |
|  | **Land use total quantity failure policy** | **Low degree failure policies** (parameter adjustment range is 0-0.33) | 13-D-W-+ 38-I-S-+ | Unused land→ Pasture | Simulation ＞Reality | Policy is inefficient in practice. |
|  |  |  | 02-D-S-+ | Lake and pond→ Cultivated land | Simulation ＞Reality |  |
|  |  |  | 01-D-S- + 17-D-W- -  21-D-W- - 28-D-W- -  31-D-W- - 43-I-S-+ | Wetland→ Cultivated land | Simulation ＞Reality |  |
|  |  |  | 39-I-S- + 43-I-S- + | Forest→ Other construction land | Simulation ＜Reality | Policy is excessive in practice. |
|  |  |  | 33-D-W- - 39-I-S-+ | Cultivated land→ Other construction land | Simulation ＜Reality |  |
|  |  |  | Policy absent | River→ Wetland | Simulation＜Reality | Policy is absent in practice. |
|  |  | **Medium degree failure policies** (parameter adjustment range is 0.33-0.66) | Policy absent | Cultivated land→ River | Simulation ＜Reality | Policy is absent in practice. |
|  |  |  | Policy absent | Cultivated land→ Lake and pond | Simulation ＜Reality |  |
|  |  | **High degree failure policies** (parameter adjustment range is 0.66-1.00) | 03-D-S- + 13-D-W-+  18-D-W-+ 25-D-W-+  38-I-S- + | Unused land→ Forest | Simulation ＞Reality | Policy is inefficient in practice. |
|  |  |  | 23-D-W- - 24-D-W- -  28-D-W- - 30-D-W- -  37-D-W- - 38-I-S-+  49-I-W- - | Unused land→ Cultivated land | Simulation ＞Reality |  |
|  |  |  | 39-I-S-+ | Cultivated land→ Land for transport | Simulation ＜Reality | Policy is excessive in practice. |
|  |  |  | Policy absent | Forest→ Unused land | Simulation ＜Reality | Policy is absent in practice. |
|  |  |  | Policy absent | Cultivated land→ Unused land | Simulation ＜Reality |  |

**Additional information:**

Here, we present the results of four failed parameter combination adjustments and the corresponding statistical table of land use simulation results. Although after these several adjustments, the difference between the simulation results and the actual results is not within the accuracy range. Each time the results of the adjustment was better than the last time to get closer to the real status, and every parameter adjustment was based on the results of the last adjustment. Therefore, through the process of four failed parameter combination adjustments, it can help to see the specific parameter adjustment idea and method.

**Table S7.**

The first round of parameter combination adjustment as the conversion cost matrix parameters input to the GeoSOS-FLUS software.

**Table S8.**

Comparison of results under the parameter combination of the first scenario simulation.

| **Land use type** | | **Actual land area in 2000 (km^2^)** | **Actual land area in 2015 (km^2^)** | **Simulated land area after the first adjustment in 2015 (km^2^)** | **Absolute error (km^2^)** | **Systematic error (%)** |
| --- | --- | --- | --- | --- | --- | --- |
| **Natural spaces** | **Forest** | 2750.79 | 2695.16 | 2730.90 | 35.74 | 1.33 |
|  | **Pasture** | 101.03 | 100.82 | 101.03 | 0.21 | 0.20 |
|  | **River** | 633.68 | 608.71 | 633.68 | 24.98 | 4.10 |
|  | **Lake and pond** | 2737.29 | 3042.88 | 2721.21 | -321.68 | **-10.57** |
|  | **Wetland** | 99.33 | 101.36 | 98.43 | -2.93 | -2.89 |
|  | **Unused land** | 7.44 | 12.69 | 6.60 | -6.09 | **-47.97** |
| **Unnatural spaces** | **Cultivated land** | 10112.79 | 7705.93 | 8116.65 | 410.72 | **5.33** |
|  | **City and town areas** | 572.10 | 1603.23 | 1603.23 | 0.00 | 0.00 |
|  | **Rural residential land** | 1227.68 | 1458.70 | 1458.70 | 0.00 | 0.00 |
|  | **Land for transport** | 92.84 | 233.91 | 92.96 | -140.94 | **-60.26** |
|  | **Industrial land** | 145.04 | 778.12 | 778.12 | 0.00 | 0.00 |
|  | **Other construction land** | 55.90 | 194.40 | 194.40 | 0.00 | 0.00 |

**Table S9.**

The second round of parameter combination adjustment as the conversion cost matrix parameters input to the GeoSOS-FLUS software.

**Table S10.**

Comparison of results under the parameter combination of the second scenario simulation.

| **Land use type** | | **Actual land area in 2000 (km^2^)** | **Actual land area in 2015 (km^2^)** | **Simulated land area after the second adjustment in 2015 (km^2^)** | **Absolute error (km^2^)** | **Systematic error (%)** |
| --- | --- | --- | --- | --- | --- | --- |
| **Natural spaces** | **Forest** | 2750.79 | 2695.16 | 2730.79 | 35.63 | 1.32 |
|  | **Pasture** | 101.03 | 100.82 | 101.03 | 0.21 | 0.20 |
|  | **River** | 633.68 | 608.71 | 633.68 | 24.98 | 4.10 |
|  | **Lake and pond** | 2737.29 | 3042.88 | 3042.88 | 0.00 | 0.00 |
|  | **Wetland** | 99.33 | 101.36 | 98.43 | -2.92 | -2.88 |
|  | **Unused land** | 7.44 | 12.69 | 6.62 | -6.06 | **-47.80** |
| **Unnatural spaces** | **Cultivated land** | 10112.79 | 7705.93 | 7795.11 | 89.18 | 0.48 |
|  | **City and town areas** | 572.10 | 1603.23 | 1603.23 | 0.00 | 0.00 |
|  | **Rural residential land** | 1227.68 | 1458.70 | 1458.70 | 0.00 | 0.00 |
|  | **Land for transport** | 92.84 | 233.91 | 92.91 | -141.00 | **-60.28** |
|  | **Industrial land** | 145.04 | 778.12 | 778.12 | 0.00 | 0.00 |
|  | **Other construction land** | 55.90 | 194.40 | 194.40 | 0.00 | 0.00 |

**Table S11.**

The third round of parameter combination adjustment as the conversion cost matrix parameters input to the GeoSOS-FLUS software.

**Table S12.**

Comparison of results under the parameter combination of the third scenario simulation.

| **Land use type** | | **Actual land area in 2000 (km^2^)** | **Actual land area in 2015 (km^2^)** | **Simulated land area after the third adjustment in 2015 (km^2^)** | **Absolute error (km^2^)** | **Systematic error (%)** |
| --- | --- | --- | --- | --- | --- | --- |
| **Natural spaces** | **Forest** | 2750.79 | 2695.16 | 2730.42 | 35.26 | 1.31 |
|  | **Pasture** | 101.03 | 100.82 | 101.03 | 0.21 | 0.20 |
|  | **River** | 633.68 | 608.71 | 630.68 | 21.97 | 3.61 |
|  | **Lake and pond** | 2737.29 | 3042.88 | 3042.88 | 0.00 | 0.00 |
|  | **Wetland** | 99.33 | 101.36 | 101.36 | 0.00 | 0.00 |
|  | **Unused land** | 7.44 | 12.69 | 6.61 | -6.08 | **-47.94** |
| **Unnatural spaces** | **Cultivated land** | 10112.79 | 7705.93 | 7795.53 | 89.60 | 0.49 |
|  | **City and town areas** | 572.10 | 1603.23 | 1603.23 | 0.00 | 0.00 |
|  | **Rural residential land** | 1227.68 | 1458.70 | 1458.70 | 0.00 | 0.00 |
|  | **Land for transport** | 92.84 | 233.91 | 92.95 | -140.95 | **-60.26** |
|  | **Industrial land** | 145.04 | 778.12 | 778.12 | 0.00 | 0.00 |
|  | **Other construction land** | 55.90 | 194.40 | 194.40 | 0.00 | 0.00 |

**Table S13.**

The fourth round of parameter combination adjustment as the conversion cost matrix parameters input to the GeoSOS-FLUS software.

**Table S14.**

Comparison of results under the parameter combination of the fourth scenario simulation.

| **Land use type** | | **Actual land area in 2000 (km^2^)** | **Actual land area in 2015 (km^2^)** | **Simulated land area after the fourth adjustment in 2015 (km^2^)** | **Absolute error (km^2^)** | **Systematic error (%)** |
| --- | --- | --- | --- | --- | --- | --- |
| **Natural spaces** | **Forest** | 2750.79 | 2695.16 | 2730.60 | 35.43 | 1.31 |
|  | **Pasture** | 101.03 | 100.82 | 101.03 | 0.21 | 0.20 |
|  | **River** | 633.68 | 608.71 | 630.72 | 22.01 | 3.62 |
|  | **Lake and pond** | 2737.29 | 3042.88 | 3042.88 | 0.00 | 0.00 |
|  | **Wetland** | 99.33 | 101.36 | 101.36 | 0.00 | 0.00 |
|  | **Unused land** | 7.44 | 12.69 | 6.63 | -6.06 | **-47.76** |
| **Unnatural spaces** | **Cultivated land** | 10112.79 | 7705.93 | 7795.34 | 89.41 | 0.48 |
|  | **City and town areas** | 572.10 | 1603.23 | 1603.23 | 0.00 | 0.00 |
|  | **Rural residential land** | 1227.68 | 1458.70 | 1458.70 | 0.00 | 0.00 |
|  | **Land for transport** | 92.84 | 233.91 | 92.91 | -140.99 | **-60.28** |
|  | **Industrial land** | 145.04 | 778.12 | 778.12 | 0.00 | 0.00 |
|  | **Other construction land** | 55.90 | 194.40 | 194.40 | 0.00 | 0.00 |
